
JOURNAL INFORMATION
==============================
NLM Title Abbreviation: ScientificWorldJournal
Journal ID: ScientificWorldJournal
NLM Journal ID: 101131163
Journal ID: TSWJ

The Scientific World Journal

ISSN: 2356-6140
EISSN: 1537-744X
Publisher: Wiley

ARTICLE INFORMATION
==============================
PMCID: PMC5849130
PMID: 18836667
DOI: 10.1100/tsw.2008.132
Article ID: 209081
Article version: 1
Subjects: Directions in Science

Can We Rescue Salivary Gland Function after Irradiation?

Feng Jielin 1 2
Coppes Robert P. 1 3 *
1 Department of Cell Biology, Section Radiation and Stress Cell Biology, University Medical Center Groningen, University of Groningen, The Netherlands
2 Department of Cell Biology, Section Stem Cell Biology, University Medical Center Groningen, University of Groningen, The Netherlands
3 Department of Radiation Oncology, University Medical Center Groningen, University of Groningen, The Netherlands
*Robert P. Coppes: r.p.coppes@med.umcg.nlAcademic Editor: Martin Goette

Publication date: 2008
Electronic publication date: 2008 Oct 3
Volume: 8
First page: 959
Last page: 962
Received 2008 Jul 9; Revised 2008 Sep 5; Accepted 2008 Sep 10
Copyright: Copyright © 2008 Jielin Feng and Robert P. Coppes.
Copyright year: 2008
License: This is an open access article distributed under the Creative Commons Attribution License, which permits unrestricted use, distribution, and reproduction in any medium, provided the original work is properly cited.
License URL: https://creativecommons.org/licenses/by/3.0/

Keywords: stem cell therapy, submandibular gland, radiotherapy, xerostomia

==============================
Directions in Science
*TheScientificWorldJOURNAL* (2008) 8, 959–962
ISSN 1537-744X; DOI 10.1100/tsw.2008.132

TheScientificWorldJOURNAL
www.thescientificworld.com

### Can We Rescue Salivary Gland Function after Irradiation?

Jielin Feng[1,2] and Robert P. Coppes[1,3,*]

*Department of Cell Biology, [1]Section Radiation and Stress Cell Biology and [2]Section Stem Cell Biology, [3]Department of Radiation Oncology, University Medical Center Groningen, University of Groningen, The Netherlands*

**E-mail**: r.p.coppes@med.umcg.nl

*Received July 9, 2008; Revised September 5, 2008; Accepted September 10, 2008; Published October 3, 2008*

---

**KEYWORDS:** stem cell therapy, submandibular gland, radiotherapy, xerostomia

---

Every year, approximately 500,000 new patients are diagnosed with head and neck cancer worldwide[1]. Radiotherapy in combination with other treatment modalities plays an important role in the treatment, yielding a 5-year survival rate of approximately 50% for nonmetastatic, locally advanced diseases. Regretfully, many surviving patients suffer from clinical consequences of oral complications after radiotherapy, such as mucositis, hyposalivation, taste loss, osteoradionecrosis, radiation caries, and trismus[2].

Hyposalivation induced by exposure of the salivary gland to radiation can result in xerostomia (dry mouth syndrome), which burdens the patient with oral dryness or pain, dental caries, reduced taste and smell, increased risk for oral infections, hampered speech, and problems with food mastication[2,3].

Clinical management of xerostomia focusing on sialogogues to stimulate the remaining gland function, and/or stringent oral hygiene and saliva substitute agents to relieve the symptoms, does not sufficiently help to replace the complex substance of saliva[4,5,6]. In some cases, damage to the salivary glands can be prevented by using Intensity Modulated Radiation Therapy (IMRT)[7], cytoprotectants like Amifostine[8], prophylactic pilocarpine[9], or surgically transferring gland tissue out of the radiation field before radiotherapy[10]. However, none of these seems to be sufficient and applicable to all patients. Stem cell therapy may be an option to reduce radiation-induced damage to the salivary glands permanently.

In 2005, Konings et al.'s review[11] on *mechanism of salivary gland radiosensitivity* indicated that irreversible hyposalivation after radiation-induced damage is mainly caused by sterilization of these primitive glandular stem cells, preventing replenishment of aged saliva-producing cells. Bone marrow–derived cells (BMCs) have been suggested as an easy, accessible source for multipotent stem cells that could potentially transdifferentiate and/or repair other nonhematopoietic organs[12,13,14,15]. Recently, Lombaert et al. described in two papers[16,17] that BMCs can be mobilized and home to the damaged salivary gland after irradiation, and induce limited repair processes of function and morphology in both submandibular gland and blood vessels. However, this process does not involve transdifferentiation of BMCs to salivary gland cells. Therefore, we looked for a better approach using tissue stem cells since regeneration of the salivary gland originates from putative stem cells[18,19,20] residing in the ductal compartment[21,22].

*Corresponding author.
©2008 with author.
Published by TheScientificWorld; www.thescientificworld.com

### ISOLATION OF SALIVARY GLAND STEM CELLS

With the fast development of techniques in the field of cell biology, many studies have revealed that adult stem cells are potentially able to regenerate organs or tissues. From such tissues as brain[23], mammary gland[24], pituitary gland[25], retina[26], skin[27], inner ear[28], and pancreas[29], stem cells can be isolated, characterized, and cultured as *in vitro* floating sphere cultures. With slight modification, Lombaert et al. isolated stem cells from salivary glands of mice using this *in vitro* floating sphere culture system for mouse salivary gland tissue[30]. A cell suspension of mice submandibular gland was obtained by enzymatic digestion. This was transferred to defined medium containing several growth factors. Within 3 days, from the initial 2–3 × $10^6$ cells plated, ~9,000 spheres per digested submandibular gland were formed. Cells in these spheres were actively proliferating, indicating that the spheres did not originate from cell aggregation.

### CHARACTERIZATION OF SALIVARY GLAND STEM CELLS

The salivary gland origin of the spheres was indicated by a set of (immuno-)histochemical analyses showing expression of a specific salivary gland protein, such as PAS (Periodic Acid Schiff's base), CK7, CK14, and amylase[30]. The potential presence of stem cells was shown by the expression of known stem cell markers, such as Sca-1[31], c-Kit[32], and Musashi-1[33]. Remarkably, the spheres could, when put into a 3D collagen culture system, form specific salivary gland tissue structures, such as elongated ducts and $PAS^+$ acinar cells.

### SALIVARY GLAND STEM CELL TRANSPLANTATION

For transplantation studies[30], Lombaert et al. used male (Actbe-)GFP transgenic mice to track the progeny of the transplanted cells. Dissociated cells obtained from spheres cultured from male (Actbe-) GFP transgenic mice for 3 days were injected into submandibular glands of female mice, of which the salivary glands were irreversibly damaged by irradiation. Ninety days after transplantation, (immuno-) histochemical analyses indicated that in the transplanted glands, a high level of proliferation was ongoing. The morphology appeared as normal as in unirradiated glands and contained a large number of acinar cells. Quantification of the total surface area comprising acinar cells revealed a strong increase after salivary gland stem cell transplantation in most transplanted recipients. GFP expression and *in situ* hybridization for the donor Y-chromosome indicated that the newly formed acinar cells were donor derived. Furthermore, whereas nontransplanted irradiated animals hardly produce any saliva (0-5 µl/15 min), after transplantation, a clear improvement of salivary gland function was measured 90 days postirradiation, ranging from 23–70% of control flow rate (±200 µl/15 min, collected from restrained mice after pilocarpine injection).

To further characterize salivary stem cells, 300–1000 c-$Kit^+$ cells isolated by flow cytometry from 3-day cultured spheres were transplanted in the same way, which resulted in a pronounced improvement in saliva secretion (30–150% of control) of nine out of 13 animals (69%) at 120 days postirradiation. This effect is extraordinary as only two of the six salivary glands were treated, whereas all were irradiated and normally contribute to the pilocarpine-induced saliva secretion. In contrast, transplantation of 10,000–90,000 of c-Kit–negative cells results in only one complete and two minor responders (out of nine). To test *in vivo* self-renewal and repopulating potential of engrafted cells, a secondary transplantation, with c-$kit^+$ cells isolated from the primary recipients, was performed according to the same protocol[30]. Again, an amelioration of radiation-induced gland dysfunction in secondary recipients was evident.

In conclusion, we show restoration of function of irreversibly damaged mouse submandibular glands after intraglandular injection of an *in vitro* cultured c-$Kit^+$ cell population containing salivary gland stem cells. Our findings raise the prospect of clinical autologous salivary gland stem cell transplantation after radiotherapy.

Since surgical removal of salivary gland tissue during lymph node dissection prior to radiotherapy could provide stem cells to be transplanted postirradiation, transplantation of autologous salivary gland stem cells, compared to BMCs, may be a more adequate and elegant method of therapy. Indeed, we have been able to culture salispheres from both human parotid and submandibular gland, and show the presence of c-kit–expressing cells in the tissue, indicating the potential feasibility for translation to a clinical situation.

However, some questions still remain unanswered. To achieve more efficient transplantation, it is necessary to further characterize the exact cells with regenerative potential. In clinical therapy, more optimal techniques to inject the stem cells into the damaged salivary glands may increase the overall outcome. Injection into the excretory ducts of the glands seems to be optimal to get engrafted cells into the gland environment directly and with less clinical injury.

The presently described model appears readily translatable to the clinic and may lead, in the near future, to clinically applicable use of salivary gland tissue stem cells. Our data predict that transplantation of these cells will result in amelioration of the severely reduced quality of life of surviving cancer patients. Furthermore, our approach is the first proof for the potential use of stem cell transplantation to functionally rescue solid organ deficiency.

### REFERENCES

1. Jemal, A., Siegel, R., Ward, E., Hao, Y., Xu, J., Murray, T., and Thun, M.J. (2008) Cancer statistics, 2008. *CA Cancer J. Clin.* **58,** 71–96.
2. Vissink, A., Jansma, J., Spijkervet, F.K., Burlage, F.R., and Coppes, R.P. (2003) Oral sequelae of head and neck radiotherapy. *Crit. Rev. Oral Biol. Med.* **14,** 199–212.
3. Vissink, A., Burlage, F.R., Spijkervet, F.K., Jansma, J., and Coppes, R.P. (2003) Prevention and treatment of the consequences of head and neck radiotherapy. *Crit. Rev. Oral Biol. Med.* **14,** 213–225.
4. Regelink, G., Vissink, A., Reintsema, H., and Nauta, J.M. (1998) Efficacy of a synthetic polymer saliva substitute in reducing oral complaints of patients suffering from irradiation-induced xerostomia. *Quintessence Int.* **29,** 383–388.
5. Mosqueda-Taylor, A., Luna-Ortiz, K., Irigoyen-Camacho, M.E., Diaz-Franco, M.A., and Coll-Munoz, A.M. (2004) Effect of pilocarpine hydrochloride on salivary production in previously irradiated head and neck cancer patients. *Med. Oral* **9,** 204–211.
6. Johnson, J.T., Ferretti, G.A., Nethery, W.J., Valdez, I.H., Fox, P.C., Ng, D., Muscoplat, C.C., and Gallagher, S.C. (1993) Oral pilocarpine for post-irradiation xerostomia in patients with head and neck cancer. *N. Engl. J. Med.* **329,** 390–395.
7. Dirix, P., Nuyts, S., and Van den Bogaert, W. (2006) Radiation-induced xerostomia in patients with head and neck cancer: a literature review. *Cancer* **107,** 2525–2534.
8. Bourhis, J., Thephamongkhol, K., and Pignon, J.P. (2004) Randomized trials of amifostine and radiotherapy: effect on survival? *Semin. Oncol.* **31,** 62–66.
9. Burlage, F.R., Roesink, J.M., Kampinga, H.H., Coppes, R.P., Terhaard, C., Langendijk, J.A., van Luijk, P., Stokman, M.A., and Vissink, A. (2008) Protection of salivary function by concomitant pilocarpine during radiotherapy: a double-blind, randomized, placebo-controlled study. *Int. J. Radiat. Oncol. Biol. Phys.* **70,** 14–22.
10. Al-Qahtani, K., Hier, M.P., Sultanum, K., and Black, M.J. (2006) The role of submandibular salivary gland transfer in preventing xerostomia in the chemoradiotherapy patient. *Oral Surg. Oral Med. Oral Pathol. Oral Radiol. Endod.* **101,** 753–756.
11. Konings, A.W., Coppes, R.P., and Vissink, A. (2005) On the mechanism of salivary gland radiosensitivity. *Int. J. Radiat. Oncol. Biol. Phys*. **62,** 1187–1194.
12. Couzin, J. (2006) Clinical trials. A shot of bone marrow can help the heart. *Science* **313,** 1715–1716.
13. Lagasse, E., Connors, H., Al-Dhalimy, M., Reitsma, M., Dohse, M., Osborne, L., Wang, X., Finegold, M., Weissman, I.L., and Grompe, M. (2000) Purified hematopoietic stem cells can differentiate into hepatocytes in vivo. *Nat. Med*. **6,** 1229–1234.
14. Nishida, M., Fujimoto, S., Toiyama, K., Sato, H., and Hamaoka, K. (2004) Effect of hematopoietic cytokines on renal function in cisplatin-induced ARF in mice. *Biochem. Biophys. Res. Commun.* **324,** 341–347.
15. Orlic, D., Kajstura, J., Chimenti, S., Jakoniuk, I., Anderson, S.M., Li, B., Pickel, J., McKay, R., Nadal-Ginard, B., Bodine, D.M., Leri, A., and Anversa, P. (2001) Bone marrow cells regenerate infarcted myocardium. *Nature* **410,** 701–705.

16. Lombaert, I.M., Wierenga, P.K., Kok, T., Kampinga, H.H., deHaan, G., and Coppes, R.P. (2006) Mobilization of bone marrow stem cells by granulocyte colony-stimulating factor ameliorates radiation-induced damage to salivary glands. *Clin. Cancer Res.* **12,** 1804–1812.
17. Lombaert, I., Brunsting, J., Wierenga, P. Kampinga H.H., deHaan G., and Coppes R.P. (2008) Flt-3 ligand/granulocyte-colony stimulating factor/stem cell factor treatment ameliorates radiation-induced parenchymal and vascular damage in the salivary gland. *Clin. Cancer Res.*, in press.
18. Burford-Mason, A.P., Cummins, M.M., Brown, D.H., MacKay, A.J., and Dardick, I. (1993) Immunohistochemical analysis of the proliferative capacity of duct and acinar cells during ligation-induced atrophy and subsequent regeneration of rat parotid gland. *J. Oral Pathol. Med.* **22,** 440–446.
19. Takahashi, S., Nakamura, S., Suzuki, R., Islam, N., Domon, T., Yamamoto, T., and Wakita, M. (2000) Apoptosis and mitosis of parenchymal cells in the duct-ligated rat submandibular gland. *Tissue Cell* **32,** 457–463.
20. Takahashi, S., Shinzato, K., Nakamura, S., Domon, T., Yamamoto, T., and Wakita, M. (2004) Cell death and cell proliferation in the regeneration of atrophied rat submandibular glands after duct ligation. *J. Oral Pathol. Med*. **33,** 23–29.
21. Denny, P.C. and Denny, P.A. (1999) Dynamics of parenchymal cell division, differentiation, and apoptosis in the young adult female mouse submandibular gland. *Anat. Rec.* **254,** 408–417.
22. Man, Y.G., Ball, W.D., Marchetti, L., and Hand, A.R. (2001) Contributions of intercalated duct cells to the normal parenchyma of submandibular glands of adult rats. *Anat. Rec.* **263,** 202–214.
23. Reynolds, B.A. and Weiss, S. (1992) Generation of neurons and astrocytes from isolated cells of the adult mammalian central nervous system. *Science* **255,** 1707–1710.
24. Dontu, G., Abdallah, W.M., Foley, J.M., Jackson, K.W., Clarke, M.F., Kawamura, M.J., and Wicha, M.S. (2003) In vitro propagation and transcriptional profiling of human mammary stem/progenitor cells. *Genes Dev.* **17,** 1253–1270.
25. Chen, J., Hersmus, N., Van Duppen, V., Caesens, P., Denef, C., and Vankelecom, H. (2005) The adult pituitary contains a cell population displaying stem/progenitor cell and early embryonic characteristics. *Endocrinology* **146,** 3985–3998.
26. Tropepe, V., Coles, B.L., Chiasson, B.J., Horsford, D.J., Elia, A.J., McInnes, R.R., and van der Kooy, D. (2000) Retinal stem cells in the adult mammalian eye. *Science* **287,** 2032–2036.
27. Toma, J.G., Akhavan, M., Fernandes, K.J., Barnabe-Heider, F., Sadikot, A., Kaplan, D.R., and Miller, F.D. (2001) Isolation of multipotent adult stem cells from the dermis of mammalian skin. *Nat. Cell Biol*. **3,** 778–784.
28. Li, H., Liu, H., and Heller, S. (2003) Pluripotent stem cells from the adult mouse inner ear. *Nat. Med.* **9**, 1293–1299.
29. Seaberg, R.M., Smukler, S.R., Kieffer, T.J., Enikolopov, G., Asghar, Z., Wheeler, M.B., Korbutt, G., and van der Kooy, D. (2004) Clonal identification of multipotent precursors from adult mouse pancreas that generate neural and pancreatic lineages. *Nat. Biotechnol.* **22,** 1115–1124.
30. Lombaert, I.M., Brunsting, J.F., Wierenga, P.K., Faber, H., Stokman, M.A., Kok, T., Visser, W.H., Kampinga, H.H., de Haan, G., and Coppes, R.P. (2008) Rescue of salivary gland function after stem cell transplantation in irradiated glands. *PLoS ONE* **3,** e2063.
31. Welm, B.E., Tepera, S.B., Venezia, T., Graubert, T.A., Rosen, J.M., and Goodell, M.A. (2002) Sca-1(pos) cells in the mouse mammary gland represent an enriched progenitor cell population. *Dev. Biol.* **245,** 42–56.
32. Medina, R.J., Kataoka, K., Takaishi, M., Miyazaki, M., and Huh, N.H. (2006) Isolation of epithelial stem cells from dermis by a three-dimensional culture system. *J. Cell. Biochem*. **98,** 174–184.
33. Kayahara, T., Sawada, M., Takaishi, S., Fukui, H., Seno, H., Fukuzawa, H., Suzuki, K., Hiai, H., Kageyama, R., Okano, H., and Chiba, T. (2003) Candidate markers for stem and early progenitor cells, Musashi-1 and Hes1, are expressed in crypt base columnar cells of mouse small intestine. *FEBS Lett*. **535,** 131–135.

**This article should be cited as follows:**

Feng, J. and Coppes, R.P. (2008) Can we rescue salivary gland function after irradiation? *TheScientificWorldJOURNAL* **8**, 959–962. DOI 10.1100/tsw.2008.132.
